# Unraveling TRPV1’s Role in Cancer: Expression, Modulation, and Therapeutic Opportunities with Capsaicin

**DOI:** 10.3390/molecules29194729

**Published:** 2024-10-07

**Authors:** Subramanyam R. Chinreddy, Nicole Tendayi Mashozhera, Badraldeen Rashrash, Gerardo Flores-Iga, Padma Nimmakayala, Gerald R. Hankins, Robert T. Harris, Umesh K. Reddy

**Affiliations:** Department of Biology, West Virginia State University, Institute, WV 25112, USA; subramanyam.chinreddy@wvstateu.edu (S.R.C.); nmashozhera@wvstateu.edu (N.T.M.); brashrash2@wvstateu.edu (B.R.); juan.iga@wvstateu.edu (G.F.-I.); padma@wvstateu.edu (P.N.); ghankins@wvstateu.edu (G.R.H.); harrisro@wvstateu.edu (R.T.H.)

**Keywords:** TRPV1, cancer pain, signaling pathways, cancer therapy, capsaicin

## Abstract

Cancer is a global health challenge with rising incidence and mortality rates, posing significant concerns. The World Health Organization reports cancer as a leading cause of death worldwide, contributing to nearly one in six deaths. Cancer pathogenesis involves disruptions in cellular signaling pathways, resulting in uncontrolled cell growth and metastasis. Among emerging players in cancer biology, Transient Receptor Potential (TRP) channels, notably TRPV1, have garnered attention due to their altered expression in cancer cells and roles in tumorigenesis and progression. TRPV1, also known as the capsaicin receptor, is pivotal in cancer cell death and pain mediation, offering promise as a therapeutic target. Activation of TRPV1 triggers calcium influx and affects cell signaling linked to growth and death. Additionally, TRPV1 is implicated in cancer-induced pain and chemo-sensitivity, with upregulation observed in sensory neurons innervating oral cancers. Also, when capsaicin, a compound from chili peppers, interacts with TRPV1, it elicits a “hot” sensation and influences cancer processes through calcium influx. Understanding TRPV1’s multifaceted roles in cancer may lead to novel therapeutic strategies for managing cancer-related symptoms and improving patient outcomes. The current review elucidates the comprehensive role of capsaicin in cancer therapy, particularly through the TRPV1 channel, highlighting its effects in various cells via different signaling pathways and discussing its limitations.

## 1. Introduction

Cancer continues to be a major cause of death globally, with both its occurrence and death rates projected to increase in the coming years. In 2023, 10 million deaths were attributed to cancer, highlighting the urgent need to develop strategies to fully understand its mechanisms, enable early detection, and create more effective treatments [1,2,3]. The pathogenesis of cancer involves a complex interplay of genetic and epigenetic changes that interfere with cellular signaling pathways, including oncogenes, tumor-suppressor genes, and defects in DNA repair mechanisms, all of which contribute to genomic instability [4,5,6], leading to uncontrolled cell proliferation, evasion of apoptosis, and increased metastatic potential, therefore resulting in cancer progression [7,8,9].

Transient Receptor Potential (TRP) channels, particularly TRPV1, the capsaicin receptor, have emerged as a significant player in the cancer landscape due to altered expression levels in cancer cells and their involvement in regulating cell cycle advancement and programmed cell death, vital in cancer development [4,10,11]. The interplay between TRPV1 and key cancer molecular pathways, such as the Phosphoinositide 3-kinase/Protein kinase B (PI3K/AKT) and Mitogen-Activated Protein Kinase (MAPK) pathways, has been highlighted in several studies [12,13,14]. The modulation of TRPV1 expression and function has been connected to changes in the tumor microenvironment and immune responses, with implications for cancer metastasis and patient prognosis [15]. Similarly, TRPV1 expression on dendritic cells could modulate immune responses, affecting tumor growth and metastasis, highlighting its potential in developing immunotherapeutic strategies for cancer. Likewise, TRPV1 is upregulated in sensory neurons innervating oral cancers, contributing to pain and potentially affecting the tumor microenvironment.

Capsaicin, the main bioactive pungent compound in chili peppers, interacts with TRPV1, leading to cellular calcium influx and depolarization, which is significant in the context of cancer due to cell-signaling-related cell growth and cell death [16,17,18,19,20,21]. Capsaicin has been extensively studied for its anti-cancer effects, such as inhibiting cell proliferation and modulating cancer-related pain. These properties have been reported in breast, prostate, and colon cancer cells, and have been associated with triggering calcium influx and activating pro-apoptotic signaling pathways [22,23]. For instance, capsaicin’s interaction with TRPV1 has been associated with reduced tumor growth and enhanced chemosensitivity, positioning it as a promising adjunct in cancer therapy [24]. Capsaicin sensitizes cancer cells to chemotherapy drugs, thereby improving therapeutic outcomes [25]. This sensitization is linked to capsaicin’s activation of TRPV1, which influences drug uptake and apoptosis in cancer cells [26]. For example, the regulation of heat shock transcription factor 1 (HSF1) by TRPV1 has been explored as a mechanism to amplify cancer thermo-immunotherapy, providing a novel approach to cancer treatment [27].

Obtaining an in-depth understanding of the genetic, molecular, and cellular mechanisms underlying cancer, alongside exploring phytochemicals, is crucial for developing more effective therapeutic strategies [7,8,9]. For instance, insights into the molecular players involved in capsaicin therapy can be gained through advanced high-throughput sequencing, CRISPR gene editing, and personalized medicine, potentially offering new avenues for enhancing the efficacy of existing treatments and improving patient outcomes [9,28,29]. In this review, we aim to explore the multifaceted roles of TRPV1 in cancer by its modulation mediated by capsaicin. By examining the alteration of TRPV1 in sensory neurons and its implications for cancer pain and chemosensitivity, we seek to provide insights into the complex interplay between cancer biology and sensory perception. This investigation may open new pathways for developing therapeutic strategies aimed at managing cancer-related symptoms and improving patient outcomes. Exploring TRPV1’s function in cancer could result in the identification of novel biomarkers and therapeutic targets, thereby advancing cancer diagnosis and treatment.

## 2. TRPV1: Role in Cancer

The TRPV1 channel is a significant player in cancer biology, primarily due to its ability to modulate downstream signaling pathways that are crucial for immune modulation, inflammation, and cell survival [18]. This is exemplified by its involvement in the regulation of immune cells within the tumor microenvironment, demonstrating how TRPV1-expressing neurons interact with immune cells like regulatory T cells (Tregs) via signaling molecules such as Calcitonin gene-related peptide (CGRP) in a “neuroimmune crosstalk” [30,31]. This interaction is vital in maintaining gut homeostasis, but also has implications for cancer because it may influence immune evasion, a key challenge in tumor growth and metastasis [31]. Similarly, the modulation of immune cells by TRPV1 activation extends its influence beyond direct tumor cell survival, potentially altering the tumor microenvironment and affecting cancer progression through immune evasion mechanisms [32]. Furthermore, the modulation of TRPV1 influences calcium influx, impacting signaling pathways like PI3K/Akt and MAPK, which are dysregulated in cancer [21,33]. The suppression of TRPV1 activity diminishes the activation of these pro-survival pathways, therefore increasing cancer cells’ susceptibility to apoptosis, potentially inhibiting tumor growth [18].

The complex interplay of TRPV1—where its activation can either promote cancer cell survival or enhance apoptosis—highlights the complexity and relevance of targeting this channel in cancer therapy [18]. For instance, TRPV1’s role in neuroimmune interactions presents a potential therapeutic target, but the therapeutic strategies aimed at modulating TRPV1 must be carefully designed, taking into account the specific cancer type and the unique characteristics of the tumor microenvironment. For example, in cases where TRPV1 activation drives apoptotic pathways, enhancing its activity could be beneficial, while inhibition might be more effective in situations where TRPV1 supports tumor survival and immune evasion [34].

## 3. TRPV1 Dynamics in Cancer: Expression and Modulation Insights

The role of TRPV1 has been noticed in different cancers. For instance, TRPV1 is highly expressed in various aggressive tumors, while blocking TRPV1 can inhibit hyperthermia-induced calcium influx, which in turn suppresses heat shock protein (HSP70) overexpression by preventing HSF1 from translocating to the nucleus [35], suggesting that TRPV1 plays a critical role in the cellular stress response within tumor environments. Similarly, elevated TRPV1 mRNA levels in lung adenocarcinoma and squamous cell carcinoma tissues compared to control tissues suggest a potential role for TRPV1 in tumor progression [32]. However, TRPV1 is downregulated in gastric cancer tissues, which affects cellular proliferation, migration, and invasion through the modulation of calcium signaling [4]. In breast cancer cell lines, TRPV1 protein was found with two distinct patterns of expression that are correlated to estrogen receptor expression. This has significant implications for patient survival rates, and suggests it can potentially serve as a prognostic biomarker [36]. Likewise, TRPV1 is expressed in breast carcinoma tissues, offering potential insights for therapeutic strategies by targeting TRPV1 activation with capsaicin [37]. The elevated TRPV1 expression is also associated with improved clinical outcomes across various cancers, suggesting that TRPV1 upregulation could be a marker for decreased tumor proliferation and better prognosis [38]. Similarly, TRPV1 activation in colorectal cancer tissues leads to increased cytosolic Ca^2+^ concentration, which in turn reduces tumor growth and cell viability, highlighting TRPV1’s potential as a tumor suppressor in colorectal cancer through apoptosis induction [39]. TRPV1 is upregulated in sensory neurons innervating oral cancers, contributing to pain and potentially affecting the tumor microenvironment [40]. Furthermore, the IL-23/IL-17A/TRPV1 axis is crucial for mechanical pain via macrophage–sensory-neuron crosstalk in female mice, which is relevant to cancer pain management and the tumor microenvironment [41]. The receptor’s sensitization through phosphorylation and interaction with protease-activated receptor-2 (PAR2) highlights its significance in the nuanced mechanisms of cancer pain and progression [42].

Deng et al. explored the effects of a high-capsaicin diet on TRPV1 expression in gastric cancer cells, linking it to increased TRPV1 levels and altered tumor dynamics [43]. Nanoparticle-mediated TRPV1 blockade can enhance the efficacy of cancer thermo-immunotherapy, proposing a novel approach for improving therapeutic outcomes [27]. Kijima et al. discussed the potential of targeting HSF1 through TRPV1 activity modulation as a new strategy for cancer treatment, further expanding the therapeutic possibilities associated with TRPV1 [44]. Collectively, these studies underscore the multifaceted role of TRPV1 in cancer dynamics, offering insights into its dual function as both a tumor promoter and suppressor, depending on the context. The modulation of TRPV1 activity, whether through dietary compounds like capsaicin or targeted therapies, presents a promising avenue for future cancer treatments. 

## 4. The Role of TRPV1 in Cancer: Pain Management, Immune Modulation and Its Therapeutic Targets

TRPV1 influences pain management and immune modulation in cancer, making it a promising therapeutic target in conditions like irritable bowel syndrome, where TRPV1-expressing sensory fibers correlate with heightened pain perception [45]. Additionally, upregulation in cancer patients suggests a similar mechanism for cancer-induced pain. In this sense, capsaicin, a well-studied TRPV1 agonist, has shown efficacy in alleviating chronic cancer pain by desensitizing this channel, therefore influencing pain and reducing neuropeptide release from sensory neurons [46]. TRPV1 is expressed in the sensory neurons of cancer patients experiencing pain, emphasizing its role in pain intensity modulation [11].

Beyond pain, TRPV1 activation influences the inflammatory response associated with cancer pain, impacting cytokine release and exacerbating pain [47]. One of the promising mechanisms for pain relief in cancer is the inhibition of TRPV1, mitigating these inflammatory responses. TRPV1’s immune-modulatory functions are equally compelling. TRPV1 can regulate CD4+ T cell activation and pro-inflammatory properties, suggesting that its modulation could enhance the immune response against cancer [33]. Similarly, TRPV1 activation enhances tumor antigen presentation by dendritic cells, potentially boosting T cell-mediated immune responses [48]. TRPV1 activation in macrophages promotes pro-inflammatory cytokine release, shaping the tumor immune environment and potentially sensitizing tumors to immune-mediated destruction [49].

Emerging research underscores TRPV1’s therapeutic potential across various cancer types. TRPV1 upregulation is associated with decreased tumor proliferation, making it a therapeutic target that correlates with enhanced antitumor immune responses [38]. Moreover, TRPV1 activation increased the cytotoxicity and apoptosis induced by 5-Fluorouracil (5-FU) in MCF-7 human breast cancer cells. Therefore, TRPV1 can enhance the efficacy of chemotherapeutic agents, providing a synergistic approach to cancer treatment [50]. Additionally, TRPV1’s potential as a biomarker and therapeutic target in breast cancer has been elucidated, highlighting its role in inhibiting tumor growth and inducing apoptosis [36,37]. However, However HSP studies have identified the suppression of the TRPV1 channel, as a promising therapeutic target in lung and gastric cancer. [4,32]. In the context of gastric cancer, dietary capsaicin promotes gastric cancer metastasis mediated through TRPV1 [43]. In contrast, Gonzales et al. (2014) investigated the cytotoxic and antitumor effects of vanilloids in oral squamous cell carcinoma (OSCC), revealing that their effects occur independently of TRPV1 activation. Capsaicin significantly decreases OSCC cell viability, while capsazepine exhibits strong cytotoxicity, likely due to the production of reactive oxygen species (ROS) and induction of apoptosis, rather than TRPV1 activation. The results indicate that capsazepine holds potential as a therapeutic option for OSCC, with in vivo experiments showing effective tumor reduction and minimal side effects [51].

## 5. TRPV1’s Impact on the Tumor Microenvironment

TRPV1 serves a dual function within the tumor microenvironment. It can act as a tumor suppressor by inhibiting cancer cell growth, migration, and blood vessel formation, while also playing a role in regulating immune responses and inflammation. Because TRPV1 is involved in multiple pathways, it shows promise as a target for cancer therapies. However, careful modulation is required to prevent side effects such as pain or increased inflammation. Li et al. showed that nanoparticle-mediated TRPV1 blockade modulated the tumor microenvironment by regulating TGFβ-mediated fibrotic stroma and improving antitumor therapeutics and immune cell infiltration [27]. Gao et al. found a negative correlation between TRPV1 mRNA expression and immune cell infiltration in lung adenocarcinoma [32]. Nie et al. linked TRPV1 expression to decreased tumor proliferation markers and influenced tumor purity and stromal content [38]. As previously mentioned, Deng et al. demonstrated that a high-capsaicin diet affects gastric cancer metastasis through gut microbiota changes [43]. Hou et al. highlighted the therapeutic potential of TRPV1 activation in colorectal cancer [39]. Kameda et al. explored intracellular Ca^2+^’s role in hyperthermia-induced apoptosis, adding to the understanding of TRPV1’s involvement in calcium signaling pathways [52]. Capsaicin modulates the tumor microenvironment (TME) by reprogramming tumor-associated macrophages (TAMs), reducing immunosuppressive activity and fostering a hostile environment for tumor cells [53]. Capsaicin alters cytokine and chemokine expression, enhancing antitumor immunity [16,17]. Hou et al. demonstrated that capsaicin-induced TRPV1 activation triggers apoptosis in colorectal cancer cells through the calcineurin–NFAT2–p53 signaling pathway [39]. Capsaicin’s interaction with TRPV1 channels induces apoptosis in various cancer types, disrupting cancer cell metabolism and inhibiting tumor growth [32,43]. These mechanisms collectively contribute to inhibiting tumor growth and enhancing immune responses against cancer cells, highlighting capsaicin’s potential in cancer therapy.

## 6. Exploiting TRPV1 in Cancer Therapy: Overcoming Chemoresistance and Targeting Heat Shock Proteins to Enhance Treatment Strategies

TRPV1’s role in chemoresistance represents a critical area of research with potential implications for enhancing the efficacy of cancer treatments. Chemoresistance poses a significant challenge in cancer therapy, often leading to treatment failure and disease progression. Targeting TRPV1 offers a novel strategy to overcome this obstacle. For instance, TRPV1 activation increases the cytotoxicity and apoptosis induced by 5-Fluorouracil (5-FU) in MCF-7 human breast cancer cells, suggesting that TRPV1 activation sensitizes cancer cells to chemotherapy and enhances its effectiveness [50].

Capsaicin-induced TRPV1 activation disrupts calcium homeostasis and promotes apoptosis in hepatocellular carcinoma cells, indicating a potential mechanism to overcome chemoresistance [17]. Zhou et al. further supported this by showing that capsaicin enhances chemosensitivity and apoptosis in ovarian cancer cells through TRPV1-mediated calcium influx, suggesting TRPV1 activation can augment the effectiveness of chemotherapy across different cancer types [54]. Other studies have also demonstrated the ability of TRPV1 activation to increase the sensitivity of various cancer cell lines to different chemotherapeutic agents, highlighting its potential as a universal strategy against chemoresistance [55,56,57,58,59,60,61,62]. Moreover, combining TRPV1 agonists with conventional chemotherapy could potentially reduce the required doses of chemotherapeutic agents, minimizing side effects and improving patient compliance and quality of life during treatment [63]. This integrated approach underscores TRPV1 modulation as a promising avenue for enhancing cancer therapy outcomes by sensitizing cancer cells to chemotherapy.

Targeting multiple HSPs through TRPV1 modulation could disrupt various survival pathways simultaneously, offering a comprehensive strategy for cancer treatment. HSPs play a critical role in maintaining protein homeostasis, particularly under stress conditions, and are often overexpressed in cancer cells to support their survival and proliferation [64]. Targeting these proteins could destabilize cancer cells and amplify the efficacy of cancer treatments. Interestingly, capsaicin binds to the N-terminus of Hsp90, a pivotal HSP involved in stabilizing oncogenic proteins, triggering the lysosomal degradation of Hsp70, reducing the cell’s capacity to manage protein folding and stress responses [65]. Combining capsaicin with the HSP90 inhibitor 17-AAG boosted anti-cancer effects, showing that the activation of TRPV1 and blockade of HSP90 work together to fight tumors [28]. Modulating TRPV1 presents a novel approach to cancer therapy by promoting the degradation of critical HSPs, impairing cancer cells’ stress responses and promoting apoptosis. Some other studies also support this approach, showing that HSP90 and HSP70 overexpression correlates with poor prognosis in cancer [29]. The implications of TRPV1’s influence on HSPs extend to elevating the efficacy of chemotherapy and radiation, which induce stress in cancer cells [66,67]. By targeting TRPV1 to enhance HSP degradation, synergistic treatment regimens could be developed to improve patient outcomes in cancer therapy [35]. In conclusion, the interplay between TRPV1 and HSPs presents a promising avenue for cancer therapy. 

## 7. TRPV1’s Pathways for Proliferation and Inhibition 

**Figure 1:**
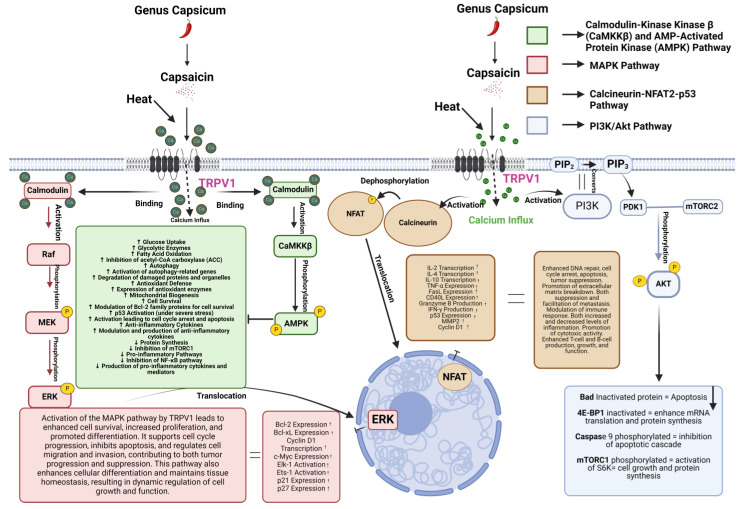
TRPV1 channel in cancer dynamics: This figure illustrates the role of the TRPV1 channel in cancer progression/inhibition and highlights the effects of capsaicin on various cancer cells. Key signaling pathways affected by capsaicin, such as the PI3K/Akt and MAPK pathways, are depicted, showcasing the molecular mechanisms involved in cancer cell modulation (illustration created using BioRender). Arrow up means upregulation, down means down regulation.

TRPV1 is a significant modulator of pathways crucial for both cancer proliferation and inhibition. One of the primary pathways affected by TRPV1 is the calcineurin–NFAT2–p53 signaling pathway; specifically, its activation through calcium signaling induces apoptosis through p53 engagement, leading to cell death and cancer suppression [39]. The activation of TRPV1 affects important cell-growth pathways, such as the PI3K/Akt and MAPK pathways (as shown in Figure 1). Similarly, studies have shown that when TRPV1 is activated by capsaicin, it increases the expression of androgen receptors in prostate cancer cells through these same pathways, which helps the cancer cells grow and survive [19].

The calcium influx provoked by TRPV1 activation represents an opportunity to control cell proliferation, thereby significantly impacting cancer progression [4]. For example, the activation of TRPV1 in the tumor microenvironment of Lewis lung carcinoma triggers the release of CGRP from sensory neurons, which contributes to tumor progression through two mechanisms: first, inducing angiogenesis by upregulating VEGF, and second, suppressing the immune response by weakening cytotoxic CD8+ T cells and reducing the activity of CD4+ T cells and NK cells. Despite the potential of targeting the TRPV1-CGRP pathway for treating cancer pain and tumor growth, the development of TRPV1 blockers for clinical use has been hindered by side effects [23]. On the other hand, TRPV1 suppression correlates with reduced gastric cancer development through the calcineurin–NFAT pathway, which is crucial for T cell activation and immune response modulation against tumors [43].

The influence of TRPV1 in HSF1 is critical in cancer. For instance, nanoparticle-mediated TRPV1 channel blockade enhances cancer thermo-immunotherapy via HSF1 modulation [27]. Additionally, TRPV1’s interaction with the PTEN pathway regulates the PI3K/Akt pathway, which holds prognostic significance in epithelial ovarian cancer by promoting cell survival and inhibiting cell proliferation [22].

## 8. Capsaicin’s Role in Tumor Suppression and Pain Management via TRPV1 Activation

TRPV1 activation through natural compounds like capsaicin presents a promising avenue for therapeutic intervention in cancer, for example, inhibiting tumor growth and metastasis through the induction of cell death pathways [16,43,46] (Table 1). It is particularly promising in challenging cancers such as triple-negative breast cancer, and in cancer-associated pain management [47]. 

Capsaicin-loaded nanoparticles selectively target cancer cells by activating TRPV1 channels, triggering calcium ion therapy within tumors [24]. This specificity and efficacy positions capsaicin as a promising component in cancer treatment strategies. Similarly, TRPV1 modulation extends to the immune system by influencing immune cell behavior in the tumor microenvironment [47]. TRPV1 activation by capsaicin also desensitizes pain pathways, offering relief to cancer patients suffering from chronic pain. Capsaicin-supported calcium phosphate nanoparticles (CAP/BSA@TCP-ZIF-8) induce tumor-specific apoptosis in HepG2 cells by releasing Ca^2+^ and capsaicin in the acidic tumor microenvironment [82], resulting in calcium overload, mitochondrial damage, and effective tumor cell death. Similarly, capsaicin modulates the TRPV1/AKT signaling pathway to reduce autophagic death and inflammation, mitigating LPS-induced acute lung injury both in vitro and in vivo [12]. Additionally, TRPV1 is activated by capsaicin in macrophages that have been pre-treated with LPS, increasing calcium levels and activating the cells. This leads to an anti-inflammatory state, similar to the M2b type, with higher levels of MHC and fewer inflammatory molecules. However, even though ERK1/2 is activated, it does not reach the nucleus [49]. Capsaicin enhances the efficacy of 5-Fluorouracil (5-FU) in HT-29 cancer cells through increased oxidative stress and apoptosis due to TRPV1. This effect, marked by elevated ROS levels, caspase activation, and mitochondrial dysfunction, is reduced by TRPV1 blockade with capsazepine, underlining TRPV1’s role in augmenting cancer therapy [83]. Moreover, capsaicin significantly amplifies the antitumor effects of Cisplatin (DDP) in tongue squamous cell carcinoma (TSCC) by promoting apoptosis [54]. Capsaicin alone reduces TSCC cell activity in a dose- and time-dependent manner, but in combination with DDP, it enhances apoptosis, inhibits proliferation, and disrupts mitochondrial function more effectively than either agent alone. This synergy is mediated through TRPV1 activation, which leads to calcium overload and triggers the calpain pathway, resulting in mitochondrial apoptosis. Collectively, these studies illustrate the broad therapeutic potential of TRPV1 modulation, with capsaicin emerging as a promising component in enhancing cancer treatment efficacy and managing inflammatory conditions through targeted activation and signaling pathways. Targeting the TRPV1 receptor may offer a non-addictive solution for managing chronic pain in cancer patients, particularly those with metastatic bone disease. Intrathecal resiniferatoxin has demonstrated long-lasting pain relief by eliminating TRPV1-expressing sensory nerves. However, the impact of TRPV1 activation on cancer progression is unclear, with studies producing varying outcomes. More research is required to clarify when TRPV1 blockade is suitable for treating cancer-related pain [84].

## 9. Capsaicin’s Role in Cell Viability and Apoptosis via TRPV1

**Figure 2:**
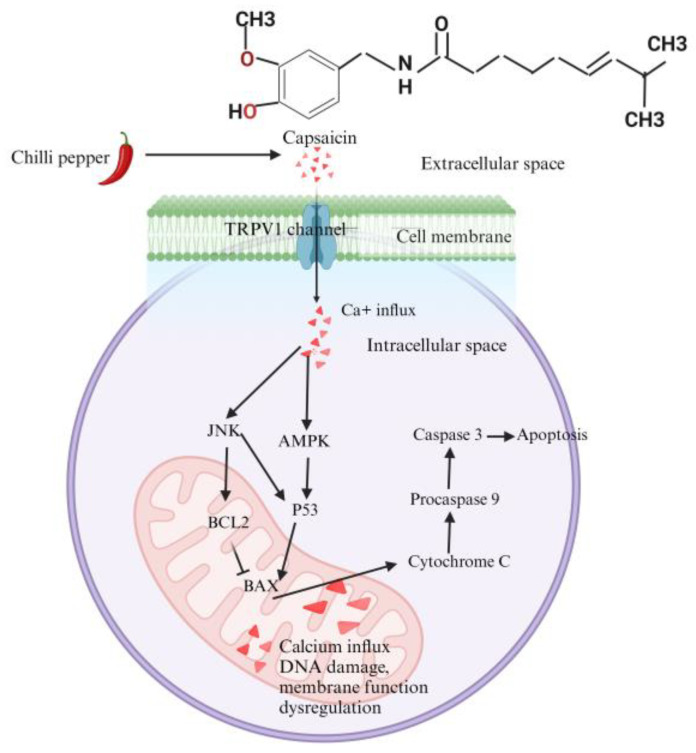
Molecular mechanism of apoptosis mediated by capsaicin-activated TRPV1 in various cancer cells. (Illustration created using Biorender.).

Capsaicin induces apoptosis, a programmed form of cell death, in various cancers, including prostate, pancreatic, colorectal, lung, breast, liver, and skin cancers [85]. This process involves structural and molecular changes such as cell shrinkage, DNA fragmentation, and chromatin condensation [86]. A key mechanism is the activation of TRPV1, a non-selective calcium channel, which plays a pivotal role in capsaicin-induced apoptosis across multiple cancer types (Figure 2) [87].

In glioma cells, capsaicin has been found to increase TRPV1 expression, leading to an influx of calcium ions (Ca^2+^), which subsequently triggers apoptosis through the activation of the p38 signaling pathway [76]. Similarly, in anaplastic thyroid cancer cells, capsaicin’s activation of TRPV1 reduces cell viability by inducing apoptosis via the intrinsic pathway. Increased intracellular calcium from TRPV1 activation causes mitochondrial overload, leading to disrupted function, elevated ROS, loss of membrane potential, and cytochrome C release [Figure 2]. This triggers caspase activation and cell death. Blocking TRPV1 or using a calcium chelator reduces capsaicin-induced apoptosis, highlighting TRPV1’s key role [77]. Amantini et al. found that TRPV1 activation in capsaicin-induced apoptosis in human urothelial cells involves the pro-apoptotic ATM protein, which is key to the DNA damage response, and Fas/CD95, which activates both intrinsic and extrinsic apoptosis pathways [78]. Apart from TRPV1, another member of the TRPV family, TRPV6, has also been implicated in capsaicin-induced apoptosis. TRPV6, a calcium-selective ion channel, helps regulate calcium homeostasis [79]. In small-cell lung cancer, capsaicin induces apoptosis by upregulating TRPV6, increasing intracellular calcium and activating the calpain pathway [79]. Another study has shown that TRPV6 overexpression in gastric cancer, post-capsaicin treatment, enhances mitochondrial permeability through Bax and p53 activation via the JNK pathway [80]. This body of research underscores the critical role of TRPV1 and TRPV6 in mediating capsaicin-induced apoptosis across different cancer cell types through calcium-dependent mechanisms.

## 10. Limitations and Future Directions in TRPV1 Research

The lack of longitudinal and cohort studies that track TRPV1 expression over time in relation to clinical outcomes across different cancers presents a limitation to the study of TRPV1’s role in cancer. These are essential to clarifying the prognostic value of TRPV1 and understanding how its expression correlates with tumor progression, metastasis, and patient survival. Furthermore, there is a pressing need for more detailed mechanistic studies that delve into the cellular and molecular mechanisms underlying TRPV1’s effects on cancer cell behavior, immune responses, and the tumor microenvironment, specifically. These studies should focus on how TRPV1 interacts with key signaling pathways, such as PI3K/Akt and MAPK, and how these interactions contribute to cancer cell survival, proliferation, and immune evasion. Another critical area for future research is the initiation of clinical trials to evaluate TRPV1-targeted therapies, either alone or in combination with existing treatments. These trials are necessary to assess the efficacy and safety of TRPV1 modulation in cancer patients and to determine the most effective therapeutic strategies. Additionally, incorporating computational biology approaches can significantly enhance our understanding of TRPV1 interactions within biological networks, predicting how TRPV1 influences various cellular processes and identifying potential new therapeutic targets. Lastly, fostering multidisciplinary collaborations between oncologists, molecular biologists, pharmacologists, and computational scientists is vital for a comprehensive study of TRPV1 in cancer. Overall, it is important to enable the integration of diverse expertise and perspectives, leading to more robust experimental designs and innovative treatment approaches to overcome current limitations and achieve breakthroughs in cancer treatment through TRPV1 modulation.

## Figures and Tables

**Table 1:** TRPV1 activity, expression, and major findings in various cancers and capsaicin’s role in cancer inhibition.

S.NO	Condition	TRPV1: Activity, Expression, Effect	Major Findings	Reference
1	Irritable bowel syndrome (IBS)	Increased TRPV1 expression in sensory fibers. Correlated with abdominal pain in IBS patients.	Increased TRPV1 expression in sensory fibers is correlated with abdominal pain in IBS patients, suggesting its role in pain modulation.	[45]
2	Gastrointestinal cancer	Various expression patterns in different GI cancers. It affected cell proliferation, migration, and invasion.	TRPV1’s role in GI cancer includes modulation of cell behavior through calcium signaling, suggesting its therapeutic potential.	[68]
3	Pan-cancer	Differential expression across cancer types. Implicated in immune cell modulation and inflammation. Modulates immune cell infiltration and inflammatory responses.	TRPV1 affects cancer cell behavior and immune responses, making it a potential target for immunotherapy across various cancers.	[33]
4	Microvascular study	Tissue-specific expression. Opposite roles in neuronal vs. smooth muscle cells. Regulates microvascular diameter.	TRPV1’s role in vascular regulation highlights its potential as a therapeutic target for vasculature-related diseases.	[69]
5	Colon cancer	TRPV1 activation by capsaicin. Induces apoptosis via PPARγ activation.	Capsaicin-induced apoptosis in colon cancer cells via TRPV1 and PPARγ activation suggests therapeutic potential.	[70]
6	Inflammatory diseases and cancer	Expressed in T cells and other immune cells. Modulates T cell responses. Influences T cell-mediated inflammation and cancer progression.	TRPV1’s role in T cell responses and inflammation highlights its therapeutic potential in cancer and inflammatory diseases.	[71]
7	Prostate cancer	Not directly addressed. TRPV1 activation by capsaicin induces apoptosis and inhibits tumor growth in prostate cancer.	Capsaicin induces apoptosis and inhibits tumor growth in prostate cancer via TRPV1, suggesting its therapeutic potential.	[72]
8	Pan-cancer	Elevated expression correlates with better clinical outcomes. Not directly measured. Negative correlation with tumor proliferation markers.	TRPV1’s upregulation is associated with decreased tumor proliferation and enhanced antitumor immune responses.	[38]
9	Bladder cancer	TRPV1 expression in urothelial cells modulates urothelial cell behavior. Influences bladder cancer progression.	TRPV1’s role in bladder cancer suggests its potential as a therapeutic target for modulating urothelial cell behavior.	[73]
10	Breast cancer	Classical and non-classical expression patterns. Estrogen-induced TRPV1 expression. Higher survival rate associated with classical TRPV1 pattern.	Classical TRPV1 expression pattern is associated with higher survival rates, suggesting its prognostic and therapeutic potential.	[36]
11	Various aggressive tumors	Overexpressed in breast, lung, hepatocellular, colorectal, and pancreatic tumors. TRPV1 blockade inhibits calcium influx and HSF1 translocation. Enhances thermotherapeutic efficacy and suppresses tumor growth.	Nanoparticle-mediated TRPV1 blockade enhances cancer therapy by modulating HSF1 pathways and improving immune infiltration.	[27]
12	Prostate cancer	Focus on androgen-independent, p53-mutant prostate cancer cells. TRPV1 activation by capsaicin inhibits growth and induces apoptosis in prostate cancer cells.	Capsaicin inhibits growth and induces apoptosis in prostate cancer cells via TRPV1 activation.	[26]
13	Different cancers	Differential TRPV1 expression levels in 12 cancers. Not directly addressed. Associated with DNA methyltransferases and mismatch repair genes.	TRPV1’s prognostic significance and association with immune microenvironment highlight its potential as a cancer biomarker.	[74]
14	Gastric cancer	High-capsaicin diet led to elevated expression of TRPV1 in gastric cancer cells. Indirectly implicated through capsaicin’s effects. Capsaicin promoted gastric cancer metastasis, partially mediated through TRPV1.	High-capsaicin diet promotes gastric cancer metastasis through TRPV1 expression modulation and gut microbiota composition changes.	[43]
15	Lung cancer (LUAD and LUSC)	Significantly higher mRNA expression in tumor tissues. Not directly addressed; focuses on mRNA expression levels. Higher TRPV1 mRNA expression is an independent risk factor for poor prognosis.	TRPV1 expression is upregulated in LUAD and LUSC and significantly negatively correlated with overall survival in LUAD patients.	[32]
16	Epithelial ovarian cancer (EOC)	Overexpressed and associated with poor prognosis. Inhibition suppressed development of EOC cells. Knockdown decreased cell viability and colony formation.	High TRPV1 expression and the combination of high TRPV1 and low PTEN expression are independent prognostic factors for EOC.	[22]
17	Colorectal cancer (CRC)	Decreased in CRC tissues compared with adjacent and normal tissues. Activation led to increased cytosolic Ca^2+^ influx and NFAT protein expression levels. Inhibited CRC growth and induced apoptosis by activating p53.	TRPV1 activation inhibits CRC cell proliferation and induces apoptosis through the calcineurin–NFAT2–p53 pathway.	[39]
18	Nasopharyngeal carcinoma (NPC)	Not explicitly detailed; study focuses on capsaicin’s effect through TRPV1. Capsaicin increased levels of IRE1, GADD153, and GRP78. Induced G0/G1-phase arrest and apoptosis in NPC-TW 039 cells.	Capsaicin induces apoptosis in NPC cells through endoplasmic reticulum stress and mitochondrial depolarization pathways via TRPV1.	[75]
19	General cancer	Not directly addressed. TRPV1 modulation through HSF1 pathways influences cancer cell survival and stress responses.	HSF1-targeted therapies involving TRPV1 modulation show potential for cancer treatment by affecting cancer cell stress responses.	[44]
20	Various cancers	IL-23/IL-17A/TRPV1 axis in immune cells modulates immune cell crosstalk and pain. Affects mechanical pain via macrophage–sensory-neuron crosstalk.	TRPV1 modulation in the IL-23/IL-17A axis influences pain and immune responses, suggesting therapeutic potential.	[41]
21	Prostate cancer	Not explicitly addressed. Capsaicin-induced effects reversed by TRPV1 antagonists. Induces androgen receptor expression and cell viability via TRPV1 activation.	Capsaicin induces androgen receptor expression and activates PI3K/Akt and ERK pathways via TRPV1, increasing cell viability in LNCaP cells.	[19]
22	Glioma cells	Capsaicin induced apoptosis via TRPV1 activation.	Apoptosis mediated through P38 MAPK activation.	[76]
23	Anaplastic thyroid cancer cells	Capsaicin-induced TRPV1 caused excess of calcium influx into mitochondria that led to mitochondrial dysfunction.	Apoptosis induced via intrinsic pathway.	[77]
24	Urothelial cancer cells	Capsaicin-activated TRPV1 induced proapoptotic ATM protein, which is vital to the DNA damage and FAS/CD95.	Both extrinsic and intrinsic pathways were induced.	[78]
25	Small-cell lung cancer	Capsaicin-induced TRPV6 activation increases intracellular calcium levels in the cytosol, leading to apoptosis.	Apoptosis induced via calpain pathway.	[79]
26	Gastric cancer	Capsaicin-activated TRPV6 increased Ca levels and affected mitochondrial permeability.	Apoptotic proteins activated through p53 via JNK pathway.	[80]
27	Ovarian cancer cells	Cisplatin-mediated TRPV1 activation leads to mitochondrial dysfunction.	Apoptosis induced by caspase-3/-8/-9 and lysosomal injury.	[81]

## Data Availability

No new data were created or analyzed in this study.
